# Research on the signaling pathway and the related mechanism of traditional Chinese medicine intervention in chronic gastritis of the “inflammation-cancer transformation”

**DOI:** 10.3389/fphar.2024.1338471

**Published:** 2024-04-18

**Authors:** Wang Yan-Rui, Yan Xue-Er, Ding Mao-Yu, Lu Ya-Ting, Lu Bo-Heng, Zhai Miao-Jie, Zhu Li

**Affiliations:** ^1^ Dongzhimen Hospital of Beijing University of Chinese Medicine, Beijing, China; ^2^ Beijing University of Chinese Medicine, Beijing, China; ^3^ Beijing Hospital of Traditional Chinese Medicine, Capital Medical University, Beijing, China

**Keywords:** chronic gastritis, “inflammation-cancer”, gastric cancer, traditional Chinese medicine, signaling pathways

## Abstract

**Objective:** The aim of this study is to uncover the traditional Chinese medicine (TCM) treatments for chronic gastritis and their potential targets and pathways involved in the “inflammation-cancer” conversion in four stages. These findings can provide further support for future research into TCM and its active components.

**Materials and methods:** The literature search encompassed PubMed, Web of Science, Google Scholar, CNKI, WanFang, and VIP, employing keywords such as “chronic gastritis”, “gastric cancer”, “traditional Chinese medicine”, “medicinal herb”, “Chinese herb”, and “natural plant”.

**Results:** Herbal remedies may regulate the signaling pathways linked to the advancement of chronic gastritis. Under the multi-target and multi-pathway independent or combined reaction, the inflammatory microenvironment may be enhanced, leading to repair of damaged gastric mucosal cells, buffering the progress of mucosal atrophic degeneration via the decrease of inflammatory factor expression, inhibition of oxidative stress-induced damage, facilitation of microvascular neovascularization in the gastric mucosa and regulation of the processes of gastric mucosal cell differentiation and proliferation. Simultaneously, the decreased expression of inflammatory factors may impact the expression of associated oncogenes and regulate the malignant proliferation of cells, thereby achieving the treatment and prevention objectives of gastric cancer through the reduction of cell metastasis and apoptosis.

**Conclusion:** Chinese medicine formulations and individual drugs can be utilised at various stages of the “inflammation-cancer” progression of chronic gastritis to prevent and treat gastric cancer in a multi-level, multi-targeted, and multi-directional fashion. This can provide guidance for the accurate application of medicines during different stages of “inflammation-cancer” transformation. New insights into the mechanism of inflammation-cancer transformation and the development of novel drugs for chronic gastritis can be gained through an extensive investigation of TCM treatment in this condition.

## 1 Introduction

Chronic gastritis is a gastric inflammation of the mucosal lining, caused by a variety of factors. It is manifest clinically by symptoms such as gastrointestinal discomfort, abdominal distension, vomiting, appetite loss, and others. Its pathologies are divided into superficial and atrophic gastritis, according to gastroscopic examinations. In the early stages of the disease, chronic superficial gastritis (CSG) or chronic non-atrophic gastritis (CNAG) are common considerations. These account for approximately 50%–85% of all gastritis cases, and present with symptoms such as gastric mucosal congestion, edema, erosion, or yellowish-white mucous exudate. Technical abbreviations will be defined upon first use. The primary pathological feature of gastritis is the degeneration of gastric epithelial cells; while most cases of superficial gastritis can be reversed, a small subset of patients with recurrent episodes may develop chronic atrophic gastritis (CAG), which is characterized by atrophy of the gastric mucosal epithelium and glands, a reduction in their quantity, and thinning of the gastric mucosa (Pellicano, 2020). Studies have shown that the development of gastric cancer is closely associated with atrophic gastritis, intestinal epithelial hyperplasia, and gastric cancer itself. Intestinal epithelial hyperplasia and heterogeneous hyperplasia are considered to be the precursors of gastric cancer (Kim et al., 2021).

Based on current research findings, gastric cancer has now risen to the fifth most frequent malignant tumour globally (Bray et al., 2018; Zhou et al., 2019), In China, the average annual incidence of gastric cancer accounts for 50% of the global incidence (Sung et al., 2021). With the gradual rise in the population of atrophic gastritis, the clinical requirements of patients with chronic gastritis have progressed from resolving basic digestive symptoms to preventing and treating chronic atrophic gastritis and gastric precancerous lesions. As a result, early intervention is highly significant to decelerate the progression of gastritis and inhibit epithelial cell lesions.

## 2 Exploring the mechanism of “inflammation-cancer” transformation

The inflammatory microenvironment is a crucial element in the development of chronic gastritis. Uncontrollable inflammation has been identified as an early instance in several types of cancer and a significant contributor to the onset and progression of tumours. When chronic inflammation persists for an extended duration, a considerable amount of inflammatory cytokines and active transmitters in the tissue’s microenvironment may cause the deactivation of activated oncogenes and tumour-suppressing genes, hastening the development of carcinomas. Moreover, uncontrolled inflammation influences the progression and conclusion of carcinomas (Colotta et al., 2009; Janssen and Henson, 2012; Baniyash et al., 2014; Greten and Grivennikov, 2019). The inflammatory environment drives tumour initiation, growth, progression and transformation, with the emergence of tumour cell infiltration. The tumour microenvironment (TME) is a key factor in tumour development and includes not only the tumour cells themselves, but also various cells such as fibroblasts, immune and inflammatory cells, glial cells and other cells in their vicinity, as well as cells in the nearby mesenchyme, microvasculature and infiltrated biomolecules (Turley et al., 2015). Inflammation has now been identified as the seventh major hallmark of cancer (Zhang et al., 2015).

The “inflammation-cancer” transformation in chronic gastritis represents the typical process of malignancy arising from long-term uncontrolled inflammation. This process follows the sequence of chronic superficial gastritis, chronic atrophic gastritis, CAG with intestinal epithelial hyperplasia, CAG with heterogeneous hyperplasia, and ultimately gastric cancer (GC). Intestinal epithelial hyperplasia with heteroplasia belongs to the stage of precancerous lesions of gastric cancer (PLGC), positioned in the middle of the cycle. To reduce the incidence of gastric cancer, it is effective to block, decrease, and reverse the chronic gastritis “inflammation-cancer transformation.” This poses a common challenge for modern Chinese and Western medicine (Jia et al., 2022).

## 3 “Inflammation-cancer”-related signaling pathways

Signalling pathways entail specific cellular responses triggered by signals that transmit information to the cell. The response triggers the transcription of relevant target genes thus regulating cellular processes such as proliferation, activation, apoptosis, and transformation. The progression of chronic gastritis usually encompasses multiple signalling pathways, including immunity, inflammation and apoptosis regulation. The development of chronic gastritis typically comprises of irregularities in several signaling pathways, such as those involved in immune responses, inflammation, and apoptosis regulation (Hu et al., 2021). The regulatory signaling pathways have a significant role in retarding or reversing the gastric inflammation and atrophic lesions, gastric precancerous lesions, or encouraging apoptosis in the cells of gastric cancer.

Chinese medicine is based on the practical experience of Chinese medicine as the main body, under the guidance of the doctrine of yin and yang and the five elements, from the dynamic perspective of the overall exploration of human physiology, pathology, pharmacology and its relationship with the natural environment, To study the law of the healthy human body and the transformation of disease and its prevention, diagnosis and treatment of the relevant content of Chinese medicine from the perspective of the development of disease as a whole, the overall exploration of the mechanism of pathological changes of chronic gastritis, so as to guide the selection of prescriptions and medication (Yang and Yang, 2021). Studies have shown the efficacy of Traditional Chinese Medicine (TCM) in reversing atrophic lesions and delaying the progression of gastritis, and the active ingredients and compounds contained in TCM monomers and combinations have a reversing effect on multidrug-resistant cells in gastritis and even gastric cancer (Shu and Yang, 2013). Moreover, TCM can also activate or inhibit relevant pathways to intervene in every stage of inflammation-cancer transition, consequently, stopping and reversing atrophic disease and precancerous gastric lesions. Simultaneously, it can activate or inhibit pathways that intervene in different stages of “inflammation-cancer transformation,” effectively preventing and reversing atrophic gastritis, slowing down the process of intestinal chemotaxis and allopatric hyperplasia, ultimately preventing gastric cancer. In this paper, we present an overview of the regulation of signal transduction during the “inflammation-cancer” stage, focusing on various signalling pathways. During the infiltration phase of the inflammatory microenvironment, the major pathways involved include TLR,NF-κB, TGFβ/Smad, Wnt/β-catenin,hedgehog, PI3K-Akt, Hippo, Notch, JAK-STAT,MAPK, etc., as the disease progresses to the stage of infiltration of the tumour microenvironment, related signalling pathways such as NF-κB, TGFβ1/Smad, Wnt/β-catenin,MAPK are involved. We also discuss the stages of inflammation-cancer transformation through the use of Chinese herbal compounds and monomers. These compounds and monomers have undergone extensive study in recent years (Supplementary Tables S1, S2), and hold potential for treating chronic gastritis, reversing gastric mucosal atrophy and precancerous lesions, and preventing gastric cancer through traditional Chinese medicine (Manikandan et al., 2010; Huang et al., 2013; Shen et al., 2013; Li et al., 2015; Wei et al., 2015; Huang et al., 2016; Lin et al., 2016; Wang et al., 2016; Yang et al., 2016; Zhao et al., 2017a; Zhao et al., 2017b; Cai et al., 2017; Zhao et al., 2017c; Li et al., 2017; Qin et al., 2017; Chen et al., 2018a; Cao, 2018; Li et al., 2018; Ma et al., 2018; Ren et al., 2018; Shi et al., 2018; Wei et al., 2018; Bai et al., 2019; Hossen et al., 2019; Hua and Xi, 2019; Ji et al., 2019; Jiang et al., 2019; Li and Li, 2019; Liu et al., 2019; Pan et al., 2019; Yan et al., 2019; Chen et al., 2020a; Sun et al., 2020a; Chen et al., 2020b; Sun et al., 2020b; Dong and Yu, 2020; Duan et al., 2020; Kang et al., 2020; Liu, 2020; Song et al., 2020; Yu et al., 2020; Zeng et al., 2020; Zhang et al., 2020; Li et al., 2021a; Yang et al., 2021a; Li et al., 2021b; Yang et al., 2021b; Cai et al., 2021; Chang et al., 2021; Li et al., 2021c; Gao et al., 2021; Hao et al., 2021; He and Guo, 2021; Ma et al., 2021; Niu et al., 2021; Qian et al., 2021; Shi et al., 2021; Tian et al., 2021; Tong et al., 2021; Wang and Li, 2021; Yan et al., 2021; Yuan et al., 2021; Ma et al., 2022a; Liu et al., 2022b; Ma et al., 2022b; Chen and Guan, 2022; Liu et al., 2022c; Hu et al., 2022; Lin et al., 2022; Tang et al., 2022; Xiang et al., 2022; Yi et al., 2022; Zhang et al., 2022; Zheng et al., 2022; Liu et al., 2023b; Dilinaer et al., 2023; Han et al., 2023; You et al., 2023).

### 3.1 TLR signaling pathway

The Toll-like receptor (TLR) is a non-catalytic receptor with wide distribution both inside and outside of human cells and performs a crucial function in inflammation, regulation of immune cells, as well as survival and proliferation. It has been discovered that a downstream signaling cascade commences following TLR activation, facilitated by bridging molecules MyD88, TIRAP, TRIF, and TRAM. These molecules polymerize on pathways such as PI3K and NF-κB, regulating intracellular kinases and gene expression, ultimately resulting in either inflammatory or antigen-specific immune responses. Technical term abbreviations are fully explained upon first use. Biased or subjective language is avoided, and the text adheres to grammatical correctness. Consistency in citation is employed. Thus, suppression of TLR expression and the downstream cascade signalling can inhibit inflammatory responses and postpone gastric mucosal lesions (Xin et al., 2010).

MyD88 is an inflammation-related molecule that bridges the downstream of the TLR signaling pathway. TCM affects the TLR pathway, which activates the downstream MyD88 factor, inhibiting the activity of inflammatory factors. As a consequence, gastric mucosal lesions reduce and intestinal chemotaxis slows down. Li found that TCM formulations such as Qinghua Yin and Jiawei Danggui Shaoyao San, as well as Bitter Ginseng and Cephalophora Polygonum, have these properties (Li et al., 2018; Jiang et al., 2019; Cai et al., 2021; Zheng et al., 2022), it is possible to affect the TLR of the CAG rat model or the TLR signaling pathway in patients with chronic gastritis. This intervention results in the inhibition of TRIFmRNA protein expression and the continuous activation of the TLR4/MyD88 signaling pathway, ultimately reducing the expression of NF-κB. Interleukin, TNF-α, and other related inflammatory factors are utilized to improve the inflammatory microenvironment of gastric tissues, reduce apoptosis, and delay the process of gastric mucosal atrophy and intestinal epithelial chemotaxis.

Chinese herbal medicines can regulate apoptosis by inhibiting oxidative stress. The effectiveness of total triterpenes in papaya was examined in an HP-infected CAG mouse model by Shi et al. (2021). The results showed that it enhanced the endogenous antioxidant system function, which inhibited HP-induced oxidative stress and inflammatory response. Additionally, it corrected lysosomal dysfunction and inflammatory activation of the TLR4/NF-κB/NLRP3 inflammasome signaling pathway. The treatment also inhibited mitochondria-mediated apoptosis, making it a promising therapy for gastritis.

### 3.2 NF-κB signaling pathway

NF-κB is a pleiotropic transcription factor with diverse regulatory functions in different directions (Yang et al., 2010). Only a small quantity of NF-κB is present in healthy cells (Hao et al., 2007), It enters the nucleus only when activated to exert its effects. Bacterial lipopolysaccharide, oxidative stress, and cytokines have an impact on its activation. Once activated, it modulates the production of cell surface receptors, pro-inflammatory cytokines, adhesion molecules, and transcription factors. NF-κB is involved in regulating genes that control various inflammatory factors that are associated with immune response, the expression and proliferation of inflammatory molecules, and anti-apoptosis (Sun and Tian, 2012). The persistent or excessive activation of NF-κB has been observed in the development of numerous inflammatory diseases. Furthermore, over-activated NF-κB is closely linked to several cancers, wherein it exhibits significantly enhanced transcriptional function following activation. This seriously hinders the normal cell signaling pathway and leads to the development of cellular carcinoma. NF-κB can promote the expression of inhibitor of apoptosis protein (IAP) during allergic reactions, autoimmune diseases, and malignant tumors, thereby inhibiting the apoptosis of tumor cells and accelerating cancer progression (Pu et al., 2006).

Traditional Chinese medicine (TCM) has the capability to hinder gastric inflammation and postpone the advancement of gastric mucosal atrophy through the mediation of the NF-κB signaling pathway. TCM formulas including Shidan granules (Hua and Xi, 2019), Weisu granules (Pan et al., 2019), and Chai Shao Liu Jun Tang (Yi et al., 2022) have been identified to be effective in this regard. It is noteworthy that these TCM formulas have been confirmed to be effective in numerous studies. It can be utilised on the gastric mucosa of CAG rat models or CAG patients to hinder the expression of subsequent pro-inflammatory factors, including interleukin, TNF-a, and NOX2, through reducing mRNA and protein levels of NF-κB signalling pathway-associated indicators. This suppression could effectively curb gastric inflammation and impede the progression of gastric mucosal atrophy.

Chinese medicine has the ability to activate the downstream STAT3 factor in treating gastric precancerous lesions. Research has shown that Banxia Xiexin Tang can impact the PLGC rat model by reducing the expression of NF-κB/STAT3 and its related pro-inflammatory factors, such as TNF-a and IL-1β, to improve the inflammatory microenvironment. This achieves the objective of treating gastric precancerous lesions (Li et al., 2017).

### 3.3 Hedgehog signaling pathway

The Hedgehog (Hh) signalling pathway facilitates intracellular transmission through cell surface cilia. It comprises three primary ligands: Shh, Ihh, and Dhh, and plays a critical role in organ development, homeostasis, and regeneration. It has been discovered that abnormally stimulated Hedgehog signalling pathway activates gastritis cancer transformation. The removal or alteration of its corresponding ligands is also significant in the development and differentiation of the gastric epithelium, maintenance of homeostasis, and the transformation of tumours (van den Brink et al., 2002; Rawadi et al., 2003; Merchant and Ding, 2017).

Chinese remedies can regulate the Hh signaling pathway, which affects the expression of related proteins and downstream inflammatory factors. Through this regulation, they create a more favourable inflammatory microenvironment in the stomach, preventing the process of mucosal damage and abnormal proliferation. The employment of Combined Formulas for Gastritis activates the SonicHedgehog signalling pathway for the treatment of the CAG rat model. This helps regulate the levels of IL-1β and GAS in serum, thus effectively improving the pathological changes in the gastric mucosal tissues of CAG rats, and slowing down the pathologic process of CAG.

Intracellular components linked to Hh signaling consist of the transcription factors Ci/Gli, serine/threonine protein kinase Fused (Fu), Fu inhibitor (SuFu), and motor proteins Costal-2 (Cos2) and protein kinase A (PKA). Ci/Gli and Fu exert a favorable regulatory function. Wei Wei Kang, a Chinese patent medicine, can reduce the expression of Gli1, Gli2, and Gli3 proteins in the gastric mucosa of rats with CAG, inhibiting excessive cell proliferation, invasion and metastasis. As a result, it improves gastric mucosal lesions and is effective in treating CAG (Chen et al., 2018a). The method of enhancing qi and activating blood protects the gastric mucosa of pre-cancerous lesions in rats by activating the Hh signaling pathway and increasing the protein amount of GAS and Shh. This results in the delay of thickening of the gastric mucosal muscularis layer and inhibition of intestinal chemotaxis. The active ingredients in use are astragali methyl glycoside and ginseng saponin under Zhao Weihan, etc.,’s PLGC rat model (Zhao et al., 2017b).

Weierning, a Chinese patent medicine, was found to decrease the serum IL-1β level and mRNA expression of inflammatory factors, including IL-6 and IL-8, in gastric tissues of the CAG rat model. This significantly reduced collagen deposition in the gastric mucosa submucosa, slowing down the apoptosis of gastric mucosal epithelial cells and, therefore, maintaining the integrity of the gastric mucosal barrier (Han et al., 2023). It also decreases the protein expression of Cdx2, Muc2, Shh, Gli1, and Smo to hinder the Hh pathway, and reverses the intestinal metaplasia of the gastric mucosa to hinder the advancement of mucosal atrophic degeneration.

### 3.4 TGF-β signaling pathway

Transforming growth factor-β (TGF-β) belongs to the group of multifunctional peptide growth factors that play a key role in regulating cell proliferation and differentiation during embryonic growth (Javelaud et al., 2011), This group comprises three isoforms, namely, β1, β2, and β3 (Barnard et al., 1993) was found to be associated with inflammatory responses and has since been identified as the cytokine most closely related to CAG. Its expression significantly increases in inflammatory and hypoxic microenvironments. TGF-β2 and β3 primarily regulate the growth and differentiation of gastrointestinal mucosa and have potential to act as coordinators of cellular renewal in the gastrointestinal epithelium. Epithelial-Mesenchymal Transition (EMT) is stimulated, resulting in epithelial tumor cells gaining a more aggressive mesenchymal-like phenotype accompanied by alterations in the expression of cell-cell adhesion molecules and metalloproteinase secretion. Such changes are implicated in the metastasis of tumor cells (Javelaud and Mauviel, 2005; Meulmeester and Ten Dijke, 2011).

Studies have demonstrated that TGF-β has the capability to convey a signal through the membrane-bound serine-threonine kinase receptor complex that is assembled as a heterodimer. Subsequently, this complex is triggered by TGF-β ligands, resulting in the phosphorylation of the SMAD family proteins (Javelaud and Mauviel, 2005). TCM can impact the TGF-β/Smad pathway and, in turn, regulate the growth and differentiation of mucosal cells. This modulation of the pathway aids in reducing inflammation in the stomach and slows down the process of inflammatory cancer transformation.

The efficacy of WeiFuchun Tablet, a Chinese patent medicine, for the treatment of CAG, has been established in studies. WeiFuchun Tablet has been found to reduce mRNA and protein levels of RUNX3, smad2, p-smad3/4, TGF-β2, and p21, while increasing bim and foxo3, which results in reduced inflammatory response (Ma et al., 2018). Zuojinwan has been observed to decrease the levels of expression of TGF-β1 and PI3K within the CAG rat model. Additionally, it inhibits the phosphorylation levels of downstream signals Akt and mTOR, regulating the growth and differentiation of gastrointestinal mucosa and ultimately slowing the progression of CAG (Tong et al., 2021).

Traditional Chinese medicine has a significant role in inhibiting the proliferation of allopatric cells and delaying the onset of gastric precancerous lesions. Shuang Pu San, a Chinese patent medicine, has the capability to decrease the expression of TGF-β1, Bcl-2, P53, and other factors in gastric mucosal cells of rats suffering from CAG. This results in the increase of Smad3 expression, which inhibits the proliferation of CAG cells and promotes the repair of gastric mucosal damage. As a result, the progression of gastric mucosal cell carcinogenesis is slowed down (Tang et al., 2022). The ancient Chinese medical formulas of Yiguanjian, Banxia Xiexin Tang, and Huanglian Wendan Tang demonstrate significant potential to inhibit TGF-β/Smad pathway activity in CAG rats or patients. This leads to a reduction in the expression of Smad protein, ultimately reversing the gastric mucosal lesions and mitigating the inflammatory reaction. Importantly, these effects have been observed to inhibit the progress of atrophic gastritis (Li et al., 2021a; He and Guo, 2021; Lin et al., 2022).

Traditional Chinese medicine can effectively inhibit the transformation of mucosal cells into cancer cells and reverse early cancerous changes. The Chinese patent medicines Qifang Wei Tong Granules and Wei Pi Xiao can impede the TGF-β1/Smad signalling pathway. They enhance the expression of E-Cadherin and curtail the expression of ZEB2 and Vimentin. This interference restrains the conversion of gastric mucosal cells into gastric glandular carcinomas, thus deferring the transit of gastric pre-cancerous lesions to gastric carcinomas. This efficacious action helps reverse the early stage of carcinomatosis (Cai et al., 2017; Yuan et al., 2021).

### 3.5 Wnt signaling pathway

The Wnt signalling pathway is a complex network that comprises various key components, including WNT-secreted proteins, β-catenin, and APC. β-catenin plays a crucial role as a downstream transduction pathway mediator in the Wnt pathway. Wnt/β-catenin is a fundamental regulatory factor for embryonic development and adult tissue homeostasis. Research evidence demonstrates that β-catenin degradable complexes, such as GSK-3β and APC, are responsible for maintaining the normal physiological state of cells. When the β-catenin degradable complex is disrupted, it is linked to the development of various diseases, including cardiovascular diseases and tumours. Mutations in the protein result in the activation of the Wnt pathway in human cancers, which promotes the process of cancer transformation (Liu et al., 2022a), Additionally, β-catenin mutation is a common cause of Wnt pathway activation in gastric cancer (Clements et al., 2002), In 2002, APC mutation and classical mutation were identified as significant pathways for gastric cancer transformation (Morin et al., 1997; McCleary et al., 2016). Overactivation of the typical Wnt pathway has been observed in 30%–50% of gastric cancer tissues and cell lines (Clements et al., 2002; Hanaki et al., 2012). Thus, the inhibition of metastatic activity in gastric cancer cells can be achieved through disruption of Wnt signaling (Hanaki et al., 2012) while promoting Epithelial-Mesenchymal Transition (EMT) for the preservation of epithelial cells and adhesion junctions (Heuberger and Birchmeier, 2010).

Chinese medicine can retard the advancement of atrophic gastritis and trigger apoptosis in gastric cancer cells through the regulation of the Wnt pathway. Studies have demonstrated that Shengyang Yiwei Tang and Bai Qiu Li Chun have a positive impact on the mouse model of chronic gastritis. The combination inhibits abnormal activation of the Wnt/β-catenin signalling pathway, thereby preventing excessive cell proliferation and differentiation, epithelial-mesenchymal transformation and reducing inflammation in gastric tissue. As a result, it provides protection to the gastric tissue (Liu et al., 2022b; Dilinaer et al., 2023).

The Chinese herbal formulas, Weiwei Decoction and Jianpihuayu Jiedu Decoction, have demonstrated efficacy in CAG rat models by reducing the expression of Wnt/β-catenin, NF-KB, NOX2, COX-2 and other inflammatory factors while increasing the expression of GSK-3β. This intervention can significantly decrease the level of gastric mucosal degeneration and inflammation in a rat model of CAG. In addition, it can enhance the regeneration of gastric mucosal capillaries, leading to the restoration of damaged gastric mucosa and eventual improvement in gastric acid secretion function. Studies indicate that baicalein has the ability to inhibit the activation of the Wnt/β-catenin pathway in gastric cancer cells. This leads to a reduction in the proliferation of SGC-7901 cells and induces their apoptosis, ultimately delaying the progression of gastric cancer (Wang et al., 2016).

### 3.6 MAPK signal pathway

MAPKs are a widely occurring serine/threonine protein kinase pathway in mammalian cells. It encompasses four distinct subpathways, namely, c-Jun N-terminal kinase (JNK), extracellular signal-regulated kinase (ERK), ERK5, and P38 mitogen-activated protein kinase (P38 MAPK). This pathway is crucial for various cellular processes such as cell proliferation, differentiation, apoptosis, and stress response (Kyriakis and Avruch, 2012). In recent years, the role of MAPK in the occurrence and development of digestive diseases has garnered significant attention. P38MAPK has been identified as promoting the aggregation and activation of white blood cells, playing a crucial role in the regulation of inflammatory response (Cuadrado and Nebreda, 2010; Chen et al., 2013a; Chen et al., 2013b), Conversely, abnormal activation of the MAPK/ERK signaling pathway can lead to the loss of apoptosis and differentiation, thus initiating abnormal cell proliferation and malignant transformation (Li and Yu, 2020). Therefore, controlling this pathway is a significant idea for treating or preventing gastric inflammation, precancerous gastric lesions, the malignant phenotype of gastric cancer, and other related processes.

Traditional Chinese medicine has the ability to inhibit inflammation and reduce gastric mucosal damage through the regulation of the MAPK pathway. Evodiine (Yang et al., 2021a) can activate the MAPK pathway and suppress IL-8 production in AGS cells, and thus serves as a potential therapy for defending against HP-induced gastritis symptoms. Andrographolide (Shen et al., 2013) has been shown to exert an effect in mice with gastritis by inhibiting extracellular signal-regulated kinase (ERK), regulating the MAPK pathway, and inhibiting the activation of inflammatory factors. This results in a reduction of inflammatory response, as well as the inhibition of abnormal cell proliferation and malignant transformation. Astragaloside was found (Li et al., 2015) to act on CAG rat models, inhibiting the production of LPS-induced inflammatory factors related to GES-1 cells. It achieves this by inhibiting the activation of the MAPK signaling pathway, thereby producing anti-inflammatory effects. Simultaneously, there is a reduction in the levels of NO and iNOS alongside an increase in SOD activity. This promotes the body’s ability to clear oxygen free radicals and inhibit tissue lipid peroxidation, thus delaying the development of gastric mucosal lesions.

Traditional Chinese medicine can also affect the MAPK pathway, thus potentially delaying or reversing the progression of gastric precancerous lesions. Studies have found that (Wei et al., 2015; Liu et al., 2022c), Jia Wei Shashen Maidong Decoction and Yi Qi Hua Yu Jie Du Prescription had an inhibitory effect on the activation of the MAPK signalling pathway in CAG rats. It is important to note that definitions for technical term abbreviations used in this text can be found in the beginning of the report. Thus, inhibiting the expression of EGF and EGFG, and the abnormal activation of the EGFR/MAPK cellular signaling pathway, improves the gastric mucosal atrophic lesions, thereby inhibiting or reversing the process of gastric precancerous lesions. Chinese patent medicine He Wei Fan Liu Kang (Wang and Li, 2021) has the ability to inhibit the expression of the p38MAPK protein in the gastric mucosa and COX-2 mRNA in gastric mucosa cells in BRG rats. This successfully reduces the inflammatory damage caused by the reflux fluid to gastric mucosa, hence delaying the progression of precancerous lesions in the gastric mucosa.

### 3.7 JAK/STAT signal pathway

The JAK/STAT signalling pathway is among the three acknowledged proinflammatory signalling pathways, with widespread existence across various tissues of the body. This pathway carries out several biological processes, which include cell proliferation, differentiation, migration, apoptosis, and immune regulation. Research has indicated that this specific pathway plays a crucial part in gastrointestinal tumours and inflammatory disorders (Wu et al., 2016). The activation of the JAK/STAT pathway sustains the elevated expression of inflammatory cells and factors, continually propelling the pathological transformations of CAG. Additionally, extensive research has revealed that this pathway is widely activated in gastrointestinal tumours, promoting their onset and progression (Wilmes et al., 2021). Consequently, inhibiting and obstructing the JAK/STAT signalling pathway is a significant consideration for treating CAG and preventing GC.

Chinese traditional medicine interventions can impact downstream inflammatory factors, resulting in the inhibition of stomach inflammation and delayed mucosal atrophic changes. Furthermore, these interventions can prevent intestinal metaplasia through the inhibition of the JAK/STAT signaling pathway. Empirical evidence suggests that Combined Formulas for Gastritis and DangguiShaoyao powder can effectively inhibit JAK1/STAT3 signaling pathways. The levels of pro-inflammatory factors TNF-α, IL-6 and IL-8 and mRNA in JAK1 and STAT3 in the serum and gastric mucosa of rats decrease, whereas the anti-inflammatory factor IL-10 level increases, leading to an improvement in the pathological changes of gastric mucosa in CAG rats (Tian et al., 2021; Liu et al., 2023b). Xiangren Weishu Decoction can impede the JAK2/STAT3 signaling pathway, which reduces the levels of P-JAK2, JAK2, STAT3, IL-8, and IL-1β in serum, as well as their expression in gastric mucosal tissue. Consequently, the pathological scores of the gastric body and antrum mucosa in rats are lowered, and the gastric mucosal damage of rats with non-atrophic gastritis is somewhat repaired (Tang et al., 2022).

Coptis and Pinellia are frequently combined in Chinese medicine to treat atrophic gastritis. Experimental results (Xiang et al., 2022) demonstrate that their primary components can diminish the concentration of serum inflammatory factors in CAG rats, hampering the JAK2/STAT3 pathway and angiogenesis pathway. This leads to the recovery of gastric mucosa morphology and prevents malignant evolution.

Chronic atrophic gastritis often presents high expression levels of STAT3. External factors can stimulate the body, leading to overactivation of JAK and subsequently resulting in the overactivation of STAT3. This overactivation can cause a disturbance in gene expression regulation, which is led by c-Myc. Such disturbance can lead to abnormal cellular metabolic mechanisms that promote cell carcinogenesis and may even result in the transformation of gastrointestinal tumors (Xu et al., 2022). Chinese patent medicine (Sun et al., 2020b) Zhitong Shun Qi capsule can reduce the levels of IL-1β and IL-6 in serum and the mRNA and protein expression levels of JAK1, STAT3 and c-Myc in gastric tissue, increase the mRNA and protein expression levels of SOCS-3 in gastric mucosal tissue, which improves the pathological changes of chronic atrophic gastritis and inhibit the carcinogenic process.

### 3.8 PI3K-Akt signal pathway

The PI3K-Akt pathway is a critical signaling pathway that maintains fundamental cell functions. Akt is a serine/threonine protein kinase located downstream of PI3K in the intracellular signal transduction system. This pathway can regulate cell proliferation, growth, size, metabolism, and activity. Recent studies have demonstrated its general activation in human cancer (Alzahrani, 2019). Tyrosine kinase receptors (RTKs) undergo phosphorylation and activation in the presence of several ligands, including growth factors and hormones. Subsequent activation of the PI3K signal and downstream phosphorylated Akt result in activation of their kinase activity. This plays a significant role in regulating gastric mucosa renewal, apoptosis and the cell cycle.

Traditional Chinese medicine can regulate the PI3K-Akt pathway, improving gastric mucosal atrophy. Experiments have confirmed (Yan et al., 2019) that JianPi YiQi prescription inhibits the PI3K-Akt signaling pathway, increasing GAS and PGE2 in rat serum and suppressing PI3K and Akt protein and gene expression in gastric tissue. Consequently, JianPi YiQi prescription significantly improves gastric mucosal atrophy in CAG rats.

PTEN is a vital tumor suppressor gene involved in the PI3K/Akt pathway and participates in P13K/Akt signal transduction. It plays a critical role in cell cycle regulation, affecting several downstream factors and preventing apoptosis. Anwei Decoction is an efficient medication for treating chronic gastric conditions, as evidenced by its ability to affect CAG rats through its interaction with the cell growth control networks of PTEN, PI3K, PDK1, Akt, and XIAP, with PI3K/Akt as the central core (Xia and Wang, 2006; Wei et al., 2018). The P13K/Akt signal transduction and XIAP gene and protein expression were suppressed in CAG rat gastric mucosa cells, prompting apoptosis of gastric mucosa cells and enhancing the pathological morphology of gastric mucosa in rats (Duan et al., 2020). XiangSha LiuJunZi Decoction, a traditional prescription, is proficient in ameliorating the living conditions and pathological status of gastric mucosa in CAG rats. The treatment of CAG can be facilitated by the increase in PTEN expression within gastric tissue and the reduction of VEGF, Akt and PI3K.

In recent years, the role of miR-21-PTEN-PI3K/Akt pathway axis in gastric cancer has been widely recognized, which is crucial to block the development of gastric cancer in patients with chronic atrophic gastritis (Wang et al., 2018). Studies have shown that (Chen et al., 2020b) XiaoPi granules can reduce the expression of miR-21 gene and increase the expression of PTEN gene in gastric mucosal tissues of rats with precancerous gastric cancer, as well as reduce the expression levels of Akt and PI3K proteins and reverse the abnormal activation of PI3K/AKT signalling pathway, thereby improving the pathological state of PLGC.

### 3.9 Hippo and notch signal pathway

The Hippo signalling pathway is closely associated with tumour initiation and development (Pimson et al., 2016; Chen et al., 2018b). It has been implicated in the progression of gastric cancer, esophageal cancer and other tumours, and is considered to be the central signalling network of mammalian cells, playing a central role in regulating cell proliferation and controlling organ growth and regeneration. MST2, RASSF1A and SAV1 are important members of the Hippo signalling pathway and have been extensively studied in recent years. Methylation of RASSF1A is a non-invasive diagnostic indicator for gastric cancer. As an adaptor protein, RASSF1A plays a role in cancer inhibition and is involved in physiological and pathological processes such as apoptosis, motility and cycle regulation (Wang, 2023). By enhancing the binding of MST2 to LATS1/2, the activity of LATS kinase and the phosphorylation of YAP1 are regulated to inhibit the cancer-promoting effect of YAP1. Hao Xinyu (Hao et al., 2021) investigated the HuaZhuoJieDu formula and found that it could improve the mucosal condition of gastric mucosa in CAG rats and increase the expression of RASSF1A, SAV1, MST2 mRNA and protein, thus delaying cancer progression.

The Notch signalling pathway is a highly conserved signalling pathway consisting of three components: Notch receptor, Notch ligand and DNA-binding sequence CSL (CBF1/Su(H)/lag1). It plays an important role in cell differentiation, development, cell proliferation and death (Previs et al., 2015), and is also correlated with the occurrence of tumours. Studies have found that (Zhang et al., 2019) E-cad, as one of the subtypes of transmembrane proteins, plays an important role in maintaining cell morphology and regulating cell adhesion, and its low expression or absence causes tumour cells to lose contact inhibition, resulting in the infinite proliferation of tumour cells. The Notch signalling pathway increases the expression of zeb1 transcription factors to reduce the expression of E-cad. It is closely related to tumour invasion and metastasis and tumour microenvironment (Meurette and Mehlen, 2018). Traditional Chinese Medicine prescription (Bai et al., 2019) BanXia XieXin Decoction can reverse the adverse reactions of CAG in rats through Notch pathway without any adverse effects. The quantity change of Notch pathway related phenotype can significantly reduce inflammatory cell infiltration, protect the morphology of gastric mucosa, increase the thickness of gastric mucosa and the number of gastric glands, increase the expression of proliferating cell nuclear antigen mucosa, so as to prevent the transformation of CAG into early gastric cancer.

## 4 Exploration of staged pathways and medications

**FIGURE 1:**
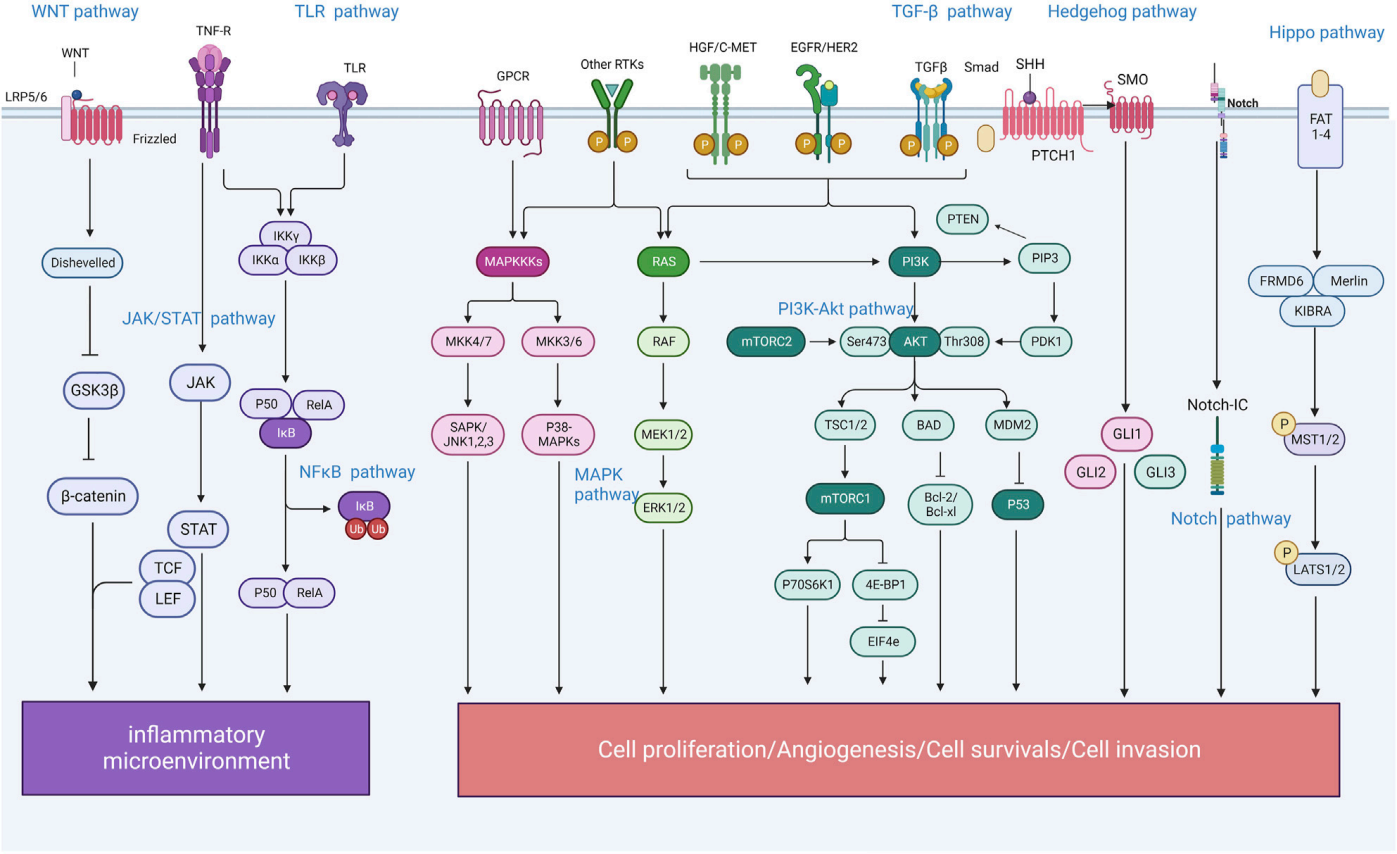
Schematic diagram of the mechanism of “inflammation-cancer transformation” of chronic gastritis regulated by traditional Chinese medicine.

**FIGURE 2:**
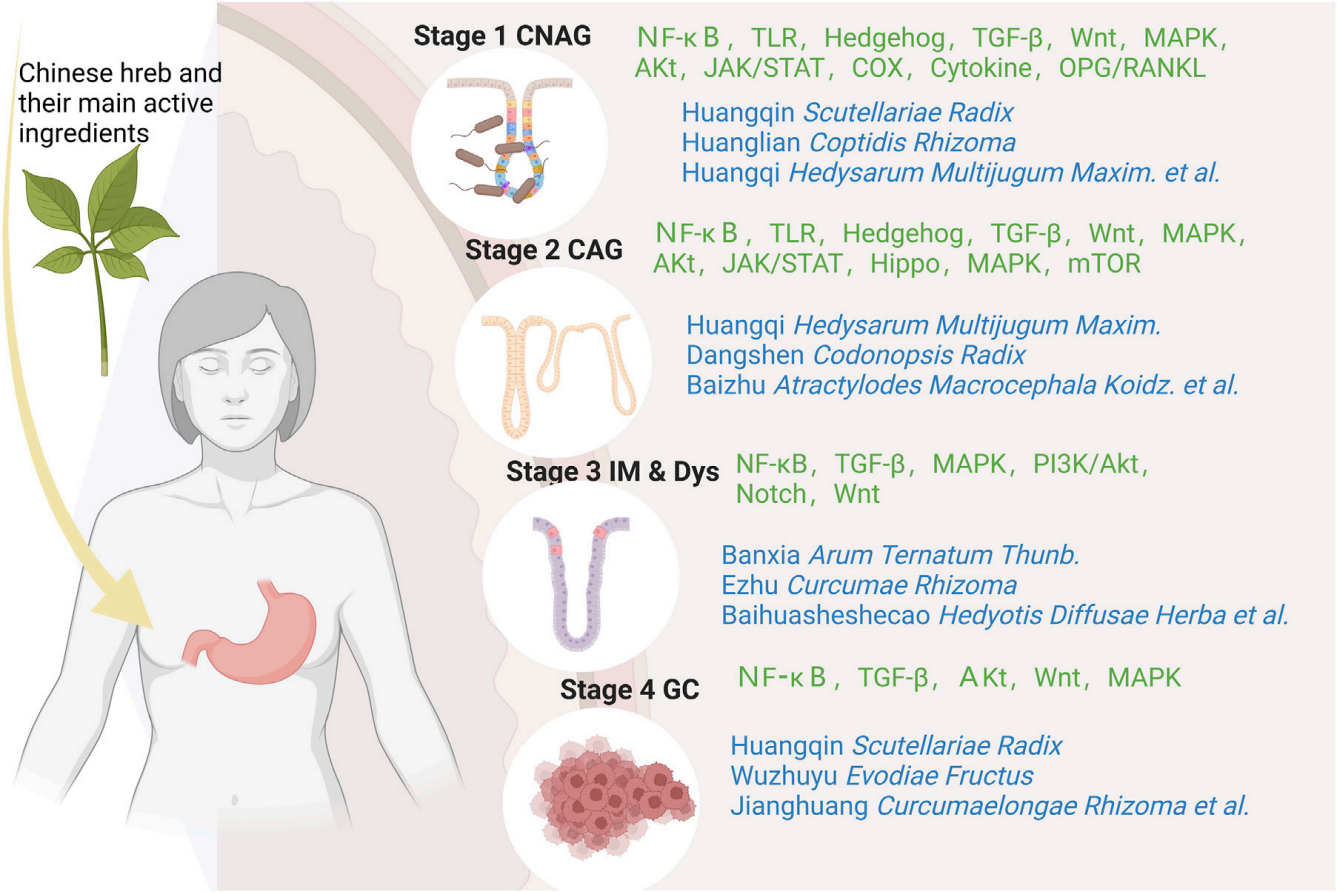
Schematic diagram of the signaling pathway of chronic gastritis “inflammation-cancer transformation” regulated by traditional Chinese medicine in stages.

According to traditional Chinese medicine, gastritis is mainly related to dietary irregularities, preference for coarse, spicy or irritating foods, tobacco and alcohol addiction, emotional and mental disorders, and improper use of medication (Kong et al., 2023). Modern studies have shown that human papillomavirus infection is one of the most important causative factors of gastritis, which is basically consistent with the traditional model described in the literature; the active ingredients of Chinese medicines can intervene in a variety of signalling pathways and have therapeutic, reversal and preventive effects on the development of gastritis at different stages. Due to the complexity of the pharmacological action of herbal medicines and the characteristics of a wide variety of constituents, which determine the diversity of their functions, the mechanism by which herbal medicines play a role in regulating signalling pathways is often a combination of several factors rather than being explained by a single pathway (Figure 1); In pre-cancerous lesions in a state of infiltration of the inflammatory microenvironment, herbal medicines are most commonly known to regulate signalling through the regulation of NF-κB, TGFβ/Smad, Hh, Notch, Hippo, Wnt/β-catenin and other signalling pathways, by inhibiting inflammatory response, oxidative stress, cell cycle regulation, endothelial vascular regeneration and other pathways, to ultimately achieve the effect of delaying or reversing atrophic changes and preventing precancerous lesions, etc. At the stage of gastric cancer, when the tumour microenvironment is infiltrated, traditional Chinese medicine is mainly used to prevent precancerous lesions by regulating NF-κB, TGFβ1/Smad, Wnt/β-catenin and other pathways. TGFβ1/Smad, Wnt/β-catenin and MAPK pathways that promote tumour cell apoptosis, inhibit angiogenesis and attenuate inflammatory responses to achieve anti-cancer effects. By illustrating different signalling pathways in the inflammatory cancer microenvironment and representative Chinese medicines (Table 1), analysing its commonly used clinical drugs and mechanism of action, it can effectively guide the different stages of treatment and precise clinical medication (Figure 2).

**TABLE 1:** Traditional Chinese medicine intervention in chronic gastritis “inflammation-cancer” transformation pathway and representative drugs.

Stage	Signalling pathways	Representative Chinese medicines	References
Inflammatory microenvironment (CNAG-IM/Dys)	NF-κB	Huangqin Scutellariae Radix., Huangqi Hedysarum Multijugum Maxim., Kushen Sophorae Flavescentis Radix., Cangzhu Atractylodes Lancea (Thunb.)Dc	Ren et al. (2018), Hossen et al. (2019), Ji et al. (2019), Cai et al. (2021)
	TGF-β/Smad	Muxiang, Aucklandiae Radix., Banxia Arum Ternatum Thunb., Huanglian Coptidis Rhizoma., PuGongYing Taraxacum mongolicum., Beishashen Glehniae Radix	Yang et al. (2016), Li et al. (2021c), Lin et al. (2022)
	TLR	Renshen, Panax Ginseng C. A. Mey., Baizhu Atractylodes Macrocephala Koidz., Kushen Sophorae Flavescentis Radix., Polygonum (Capitatum Buch.-Ham. ex D. Don)	Lin et al. (2016), Jiang et al. (2019), Cai et al. (2021)
	Hedgehog	Huangqi Hedysarum Multijugum Maxim., Sanqi Panax Notoginseng (Burk.) F. H. Chen Ex C. Chow.,Danshen, Radix Salviae., Baizhu Atractylodes Macrocephala Koidz	Zhao et al. (2017b), Zhao et al. (2017c), Li et al. (2021b)
	JAK/STAT	Huangqi Hedysarum Multijugum Maxim.,Danshen, Radix,Salviae., Banxia Arum Ternatum Thunb.,Zhike, Aurantii Fructus	Dong and Yu (2020), Tian et al. (2021), Chen and Guan (2022), Tang et al. (2022)
	Wnt/β-catenin	Guanghuoxiang Pogostemon Cablin (Blanco) Benth.,Danshen, Radix Salviae., Huangqi Hedysarum Multijugum Maxim., Banxia Arum Ternatum Thunb.,Baihuasheshecao, Hedyotis Diffuse Herba	Li and Li (2019), Yan et al. (2021), Liu et al. (2022a), Chen and Guan (2022), Dilinaer et al. (2023)
	PI3K/Akt	Ezhu, Curcumae Rhizoma., Incarvillea compacta Maxim., Banxia Arum Ternatum Thunb.,Danshen, Radix Salviae., Huangqin Scutellariae Radix	Wei et al. (2018), Hossen et al. (2019), Yan et al. (2019), Adashek et al. (2020), Zeng et al. (2020), Zhang et al. (2022)
	EGFR/MAPK	Huanglian, Coptidis Rhizoma.,Beishashen, Glehniae Radix., MaiDong Ophiopogonis Radix.,Dangshen, Codonopsis Radix.,Wuyao, Linderae Radix	Wei et al. (2015), Chen et al. (2020a), Liu et al. (2022c)
	Hippo	Huangqin Scutellariae Radix.,Huanglian, Coptidis Rhizoma.,Yinchen, Artemisiae Scopariae Herba.,Banbianlian, Lobeliae Chinensis Herba.,Baihuasheshecao, Hedyotis Diffuse Herba	Hao et al. (2021)
	Notch	Banxia, Arum Ternatum Thunb., Huangqin Scutellariae Radix., Ganjiang Zingiberis Rhizoma.,Renshen, Panax Ginseng C. A. Mey	Bai et al. (2019)
Tumour microenvironment (GC)	TGF-β/Smad	Huangqi Hedysarum Multijugum Maxim., Baizhu Atractylodes Macrocephala Koidz.,Huanglian, Coptidis Rhizoma	Yuan et al. (2021)
	Wnt/β-catenin	Huangqin Scutellariae Radix	Wang et al. (2016)
	MAPK	Wuzhuyu, Evodiae Fructus	Yang et al. (2021a), Qian et al. (2021)
	NF⁃κB	Jianghuang Curcumaelongae Rhizoma.,Ezhu, Curcumae Rhizoma	Ma et al. (2022a)

By analysing the frequency of traditional Chinese medicine, the top 30 drugs with the highest frequency were screened out (Table 2), which can suggest that in mediating the process of inflammation and cancer transformation, Coptidis Rhizoma, Poria Cocos (Schw.) Wolf., Codonopsis Radix, Atractylodes Macrocephala Koidz., Citrus Reticulata, etc. are representatives, which are widely used in inhibiting inflammation and regulating cell cycle at different stages, At the same time, in the corresponding stages, according to the different mechanisms of action, there are differences in the choice of drugs, which is considered by Chinese medicine practitioners as the largest proportion of drugs used in the treatment of this disease. According to Chinese medicine, in the treatment of this disease, the largest proportion of drugs is to clear heat and benefit qi, strengthen the spleen and dispel dampness, followed by drugs to activate the blood, disperse knots and detoxify the toxin (Kong et al., 2023), and the summary of the pathological stages can suggest the characteristics of the selection of prescriptions and the use of drugs.

**TABLE 2:** Frequency distribution of TCM for chronic gastritis “inflammation-cancer” transformation.

Serial number	TCM	Frequency/times	Serial number	TCM	Frequency/times
1	Huanglian Coptidis Rhizoma	20	16	Zhike Aurantii Fructus	9
2	Fuling Poria Cocos (Schw.) Wolf	18	17	Huangqin Scutellariae Radix	8
3	Baizhu Atractylodes Macrocephala Koidz	15	18	Zhigancao licorice	8
4	Chenpi Citrus Reticulata	15	19	Dazao Jujubae Fructus	7
5	Dangshen Codonopsis Radix	15	20	Fabanxia Arum Ternatum Thunb	7
6	Gancao licorice	15	21	Pugongying Taraxacum mongolicum	7
7	Baishao Paeoniae Radix Alba	13	22	Chaobaizhu Atractylodes Macrocephala Koidz	6
8	Banxia Arum Ternatum Thunb	13	23	Houpu Magnolia Officinalis Rehd Et Wils	6
9	Sharen Amomum Aurantiacum H. T. Tsai Et S. W. Zhao	13	24	Danggui Angelicae Sinensis Radix	5
10	Ezhu Curcumae Rhizoma	10	25	Ganjiang Zingiberis Rhizoma	5
11	Baihuasheshecao Hedyotis Diffuse Herba	9	26	Taizishen Pseudostellariae Radix	5
12	Danshen Radix Salviae	9	27	Chaihu Radix Bupleuri	4
13	Huangqi Hedysarum Multijugum Maxim	9	28	Chuanxiong Rhizoma	4
14	Muxiang Aucklandiae Radix	9	29	Peilan Eupatorium Fortunei Turcz	4
15	Sanqi Panax Notoginseng (Burk.) F. H. Chen Ex C. Chow	9	30	Renshen Panax Ginseng C. A. Mey	4

In the early stage of gastritis, Analysis of signalling pathways and related molecular mechanisms in the early stage of gastritis revealed that the conversion of CSG or CNAG to CAG mainly mediated three effects, including inhibition of inflammatory response, inhibition of oxidative stress, and promotion of apoptosis of gastric mucosal cells; it involved inflammation-related pathways represented by NF-κB, TGF-B, TLR, etc., the TCM such as Coptidis Rhizoma, Scutellariae Radix were represented. It can inhibit the expression of downstream inflammation-related factors such as IL-6, IL-8, TNF-a, NO, COX-2, etc., and enhance or activate the secretion of and enhance or activate the secretion of anti-inflammatory factors such as IL-4, IL-10, thus inhibiting inflammatory reactions, improving the inflammatory microenvironment and ameliorating the gastric mucosal lesions; for the signalling pathway represented by Wnt/β-catenin, the selection of prescriptions is based on the formulas for Jianpi Yiqi Decoction, and the traditional Chinese medicine such as Hedysarum Multijugum Maxim., Radix Salviae, etc, which can regulate the expression of β-catenin and GSK-3B, regulate the expression of endogenous oxidative factors such as PGE2 and SOD, thereby inhibiting oxidative stress, improving mucosal acidity and promoting microvascular neovascularisation; and the apoptosis-related pathway represented by PT3K/Akt, with the representative formulas such as Mieyou Decoction and Xiangsha Liujunzi Decoction, nd the traditional Chinese medicine such as Hedysarum Multijugum Maxim., Scutellariae Radix, Panax Ginseng C. A. Mey., etc, which acts on the cell growth regulation network formed by PTEN, PI3K, PDK1, Akt and XIAP with PI3K/Akt as the core, inhibits PI3K/Akt signalling and reduces XIAP gene and protein expression, thereby promoting apoptosis of gastric mucosal cells and improving the pathological morphology of the gastric mucosa.

In the stage of precancerous lesions, the transformation of medium-term CAG into PLGC or even pre-GC involves two stages of pathological progression, intestinal chemotaxis and allopatric hyperplasia, which mainly act on pathways such as NF-κB, TLR, Hippo, MAPK, etc. and mediate a variety of mechanisms, including inhibition of inflammatory response, influencing the process of cell proliferation, differentiation and apoptosis, and influencing endothelial vascular neogenesis, etc., the anti-inflammatory pathways are represented by NFκB, TLR, etc., the formula such as Shuangpu decoction and Banxia Xiexin decoction, and the traditional Chinese medicine such as Coptidis Rhizoma, Scutellariae Radix, Arum Ternatum Thunb, Panax Ginseng C. A. Mey., Angelicae Sinensis Radix,etc, Traditional Chinese medicine inhibits the activation of downstream STAT3 factors mediated in the JAK2/STAT3, NF-κB/STAT3 pathway, inhibits the expression of TRIFmRNA protein expression, and persistent activation of the TLR4/MyD88 signalling pathway leads to decreased expression of related inflammatory factors such as NF-κB, IL-6, IL-8 or TNF-a and other related inflammatory factors, increase the level of SOCS-3 mRNA and its protein expression in the gastric mucosa and slow down the process of intestinal epithelial chemotaxis and allopatric hyperplasia; Regulation of cell cycle related pathways including Hh, TGF-β, Wnt, EGFR/MAPK, PI3K-Akt, Hippo, etc., the formula such as Danggui Shaoyao powder, Banxia Xiexin decoction, Shuangpu decoction, and the traditional Chinese medicine such as Hedysarum Multijugum Maxim., Panax Notoginseng (Burk.) F. H. Chen Ex C. Chow, Curcumae Rhizoma, Arum Ternatum Thunb., Codonopsis Radix,etc, by inhibiting the expression of its cancer-related genes or promoting the expression of oncogenes, regulating the cell cycle and the process of gastric mucosal cell proliferation, differentiation, apoptosis and so on, thus inhibiting the process of malignant changes of the disease; with EGFR/MAPK and TGF-β as the representative signalling pathway, the Chinese patent medicine Weiwei Kang, Weipixiao, and the traditional Chinese medicine such as Radix Salviae, Hedyotis Diffuse Herba, Notoginseng (Burk.) F. H. Chen Ex C. Chow which can promote the mucosal vascular neovascularisation and inhibit the upper epithelium and gastric plaque, thus slowing down the process of cancer transformation.

When gastric cancer progresses to the stage of gastric cancer, at this time the positive qi is damaged, the pathological products accumulate, the cloudy poison and blood stasis caused by the long-term accumulation of poison, phlegm and dampness are long deposited in the body, and the toxic evils remain in the body for a long time, the toxicity spreads, attacks and destroys, and finally leads to the onset of GC; At this stage, treatment is mostly based on supporting positive qi and detoxifying and dispersing toxins, with the aim of attacking and dispersing the accumulation of symptoms in the body and inhibiting the progression of cancer (Liu et al., 2023a; Liao et al., 2023). TCM herbs such as Scutellariae Radix, Evodiae Fructus, Curcumaelongae Rhizoma, Hedysarum Multijugum Maxim aim to support positive qi and dispel evil spirits, and the active ingredients have been shown to be excellent at inhibiting the activity of gastric cancer cells. At this stage, traditional Chinese medicine mainly acts on NF-κB, Wnt/β-catenin, MAPK, TGF-β and other pathways to improve the inflammatory microenvironment, inhibit the proliferation and activation of gastric cancer cells, promote their apoptosis and thus slow down the progression of gastric cancer.

## 5 Summary and outlook

The inflammatory response is an important factor at all stages, and changes in the inflammatory microenvironment and the tumour microenvironment are important components of the regulatory pathways. The complexity of the tumour and inflammatory environment means that during inflammatory changes or tumour development, multiple signalling pathways overlap and form a vast network. Once activated, the different signalling pathways activate their respective positive feedback regulatory mechanisms, thereby amplifying or balancing the response. TCM mainly uses the promotion of qi and strengthening the spleen, and the promotion of diuresis and dampness as an important method throughout the disease, and combines this with the understanding of the disease development and treatment mechanism, i.e., the deficiency of qi and blood, and the transition from blooming to deficiency, which in turn is related to the accumulation of pathological products such as heat, stasis and toxins, as well as the purging of heat and dissolving turbidity, removing stasis and detoxification, According to Chinese medicine, damp-heat, toxicity and stasis are the microenvironments of inflammation and tumour that Western medicine recognises (Ma et al., 2023). Therefore, drug therapy should also focus on regulating the inflammatory microenvironment and improving the tumour microenvironment. At the same time, its kinases can activate different subtypes in combination with stage-specific pathological characteristics and regulate unique signalling pathways to achieve a precise treatment mode of “inflammation to cancer”. Stage-specific analysis allows more precise signalling and targeting for stage-specific diagnostic treatments, as well as precise and effective drug selection for specific targeted therapies and patients at specific stages.

In treating and delaying the progression of “inflammation-cancer”, TCM mainly affects the inflammatory response, proliferation, differentiation, apoptosis and transformation of gastric cells, which is of great importance in the prevention and treatment of gastric cancer (Adashek et al., 2020). Based on the analysis of the results of TCM in chronic gastritis inflammation cancer transformation, the inflammation cancer transformation therapy treatment can play a positive role in clinical practice. However, at the mechanism level, in addition to the inflammatory response, immune cells also play an important role in the tumour microenvironment, for example, TME causes a large number of regulatory T-cell (Tregs) to infiltrate and accumulate in tumour tissues, inhibiting the differentiation and maturation of effector cells (Nakamura et al., 2018), thus suppressing the immune response and evading immune surveillance, and from the perspective of the fundamental level, the surveillance function of the immune system may be the root of preventing cancer occurrence (Wang et al., 2020). At present, studies have confirmed that TCM can play an anti-tumour role by up-regulating the immune response, but the current research models are mostly cellular and animal experiments, and the research level of chronic gastritis and gastric cancer is also mostly based on the gastric cancer cell model, and there is not much research on the immune cell-related mechanism, so the therapeutic research related to the modulation of the immune mechanism by TCM to achieve the “inflammation-cancer transformation” may be a good idea. Therefore, research related to the modulation of immune mechanism by TCM to achieve “inflammation-cancer transformation” may be the focus of future research in the field of TCM. In the clinical aspect, more large-sample, multi-centre clinical trials are needed to comprehensively evaluate the clinical efficacy of TCM in “inflammation-cancer transformation”.
